# Sero-Epidemiological Study of Hepatitis E Virus among Thalassemia as High Risk Patients: A Cross-Sectional Survey in Jahrom, Southern, Iran

**DOI:** 10.5539/gjhs.v8n9p245

**Published:** 2016-01-31

**Authors:** Abdolreza Sotoodeh Jahromi, Abbas Ahmadi-vasmehjani, Hassan Zabetian, Hossein Hakimelahi, Alireza Yusefi, Mohammad Sadegh Sanie, Somayehsadat Talebnia Jahromi, Masoud Ghanei, Abdolali Sapidkar, Saiedeh Erfanian, Abdolhossien Madani, Farshid Kafilzadeh, Mohammad Kargar, Mohammad Hojjat-Farsangi

**Affiliations:** 1 Zonoses Research Center, Jahrom University of Medical Sciences, Jahrom, Iran; 2 Research Center for Social Determinants of Health, Jahrom University of Medical Sciences, Jahrom, Iran; 3 Research Center for Social of Determinants on Health Promotion, Hormozgan University of Medical Sciences, Bandarabbas, Iran; 4 Department of biology, Jahrom branch, Islamic Azad University, Jahrom, Iran; 5 Department of Oncology-Pathology, Immune and Gene Therapy Lab, Cancer Center Karolinska (CCK), Karolinska University Hospital Solna and Karolinska Institute, Stockholm, Sweden

**Keywords:** Hepatitis E Virus (HEV), prevalence, thalassemia, Jahrom

## Abstract

Hepatitis E virus (HEV) could be cause of viral hepatitis in the developing countries and cause severe epidemics. According to other studies, blood transfusion as a probable route of HEV infection has been suggested. An infection with hepatitis agents such as HEV causes active liver failure in multi-transfusion patients in particular thalassemia. The purpose of this study determines the seropositivity of anti-HEV antibodies in thalassemia individuals in Jahrom. In a cross-sectional study, sera from 110 thalassemia were collected between 2013 and 2014. Enzyme-linked immunosorbent assay (ELISA) method was performed to detection of anti-HEV antibodies. Individuals’ data were collected such as, demographic and clinical, for statistical analysis. Our results show that 10% and 1.8% of the enrolled patients were HEV Ig-G and Ig-M positive antibodies respectively. In addition, there was statiscally significant difference in age groups for prevalence of anti-HEV Ig-G (P = 0.01). Also the serum levels of liver enzymes such as ALT and AST in the HEV Ig-G and Ig-M positive samples were significantly higher than anti-HEV negative samples. But there were no significant difference between sex and splenectomy with anti-HEV positive samples. The results indicate more study are needed to assess HEV screening of blood products to these patients that those have a probably risk of exposure to HEV especially in higher years old.

## 1. Introduction

Hepatitis E virus (HEV) belongs to genus Hepevirus of the Hepeviridae family that a small nonenveloped single-stranded RNA virus (Cheng et al., 2012). Infection of hepatitis agents usually leads to benign acute hepatitis, but be fulminant particularly in patients with chronic liver disease (CLD) (Ahmadi Vasmehjani, Javeshghani, Baharlou, Shayestehpour, Mousavinasab, Joharinia, & Enderami, 2015). Also this virus is the etiologic agent for liver injury in endemic regions of world (Acharya, & Panda, 2005). It is noted that HEV infection is a main health concern in developing countries such as Iran because it occur to large epidemic in endemic regions and also to sporadic forms in developed regions (Peron, Mansuy, Izopet, & Vinel, 2006).

Transmission of HEV infection occurs by the fecal-oral route and dirty water that plays an important role in transmission route (Peron, Mansuy, Izopet, & Vinel, 2006). Also in high endemic areas, vertical and transfusion of infected blood products, is able to cause this way associated hepatitis E, are other approaches for its transmission (Acharya, & Panda, 2005). Of course, risk of taking hepatitis E virus increase with age as seroprevalence of HEV infection varied with age, from 3.3%-37.5% (Taremi, Gachkar, Mahmoudarabi, Kheradpezhouh & Khoshbaten, 2007). Previous authors have indicated a relatively seroprevalence of antibody to HEV in their hemodialysis and thalassemia patients (Abdel, M. S., El-Din, & M. E., El-Din, 1998; Halfon et al., 1994; Morteza, AbdolReza, & Hamid, 2009; Sayani et al., 2014). According to this view, patients with hemophilia and thalassemia are at higher risk of transfusion-borne viruses such as hepatitis C (HCV), TTV and other viruses (Alavian et al., 2009; Ataei, Emami Naeini, Khorvash, Yazdani, & Javadi, 2012; Sara, Solhjoo, Jahromi, & Yaghobi, 2012). Therefore this high risk groups are prone to infection with other viral hepatitis especially HEV that could lead to active and severe hepatic failure as a results in these cases, elevated liver enzyme levels is reported (Gotanda et al., 2007; Jahromi & Pourahmad, 2013).

Differences in epidemiological patterns, geographical locations, age group, disease severity and other properties associated with hepatitis E infection in different regions (Aggarwal, & Naik, 2009). In Iran, HEV infection is located in a high endemic country that is a neglected problem in our region because few outbreaks, is may be a high risk of hepatitis E occurrence (Alavian, 2007). The seroprevalence of HEV is varied in different regions of Iran (Ahmad, Roya, Manoochehr, & Nooshin, 2011; Ehteram et al., 2013; Morteza et al., 2009). The rate of seroprevalence of HEV infection in high risk such as hemodialysis patients with significance elevated liver enzyme levels were 7% (Morteza et al., 2009) but this rate in thalassemia patients was different in other studies (Al-Fawaz et al., 1996; Elizee et al., 2013; Psichogiou et al., 1996).

According to these data, but the study of HEV infection seroprevalence in high risk groups have been little performed in Iran to explain the epidemiological and clinical properties of HEV infection in these individuals. Thus, the purpose of the current study investigates HEV seroprevalence in thalassemia patients in Iran and to evaluate clinical features such as age, sex, splenectomy in patients with positive and negative HEV antibodies.

## 2. Materials and Methods

### 2.1 Study Population

A cross-sectional study was carried out Coliz unit of Motahhari hospital related to the Jahrom University of Medical Science, Iran, during July from 2013 to December 2014. A total of 110 thalassemic patients were recruited for this study. Primary screening, including human immunodeficiency virus (HIV), Human T-cell leukemia (HTLV), and hepatitis B and C were performed in all individuals and with any positive result were excluded. Information related to demographic characteristics such as sex, age and splenectomy were collected. Informed consent was obtained from all participants and their parents if the patients were under 18 years of age.

### 2.2 Serological and Biochemical Laboratory Tests

Serums of the patients were freeze at -20°C to perform immunologic studies. Serum ALT and AST levels were measured using an analyzer and values higher than 50 and 40 IU/L, respectively, Immunoglobulin G (IgG) and M (IgM) antibodies against HEV were detected using ELISA methods (DIA.PRO, Diagnostic Bioprobes Srl, Italy) according to the manufacturer instructions. The cut-off value was defined using positive and negative control sera that were included in each assay.

### 2.3 Statistical Tests

Data were entered and analyzed using SPSS software version 18.1. The Chi-square test or Fisher’s exact test was used for categorical variables. Results were reported as percentages for qualitative variables. Chi-square test and t-test were done and statistical significance was established at P values of < 0.05.

## 3. Results

The individuals’ information as demographical and clinical is shown in Table 1. Our finding shows that eleven patients (10% of the registered patients) were HEV Ig-G antibody positive and only two patients were HEV Ig-M antibody positive (Table 1). Table 2 shows the age groups, sex, splenectomy for patients with positive and negative HEV antibody, as well as their levels of ALT and AST. The highest rate of HEV positive samples came from individuals who were 11 to 20 years old. The seroprevalence of Ig-G also increased with age, rising from 0% in patients below 10 years to 10% in above 10 years group. In addition, there was significant difference in age groups for prevalence of Ig-G (P =0.01) (Table 2). The serum levels of ALT and AST in the HEV Ig-G and Ig-M positive samples were significantly higher than those in the anti-HEV negative samples. But there were no significant difference between sex and splenectomy with HEV Ig-G and Ig-M positive samples (Table 2).

**Table 1:** Demographic and clinical data for the 110 thalassemia patients

Variables	N	%	Mean±SD
**Age groups**			15.2±6
≤ 10	24	22.7	
20-Nov	69	70.9	
>20	17	6.4	
**Gender**			
Male	51	46.4	
Female	59	53.6	

**Splenectomy**			
Yes	49	44.5	
No	61	55.5	
**ALT (IU/L)**			21.5±22.1
**AST (IU/L)**			19.3±12.9
**HEV Ig-G**			
Positive	11	10	
Negative	99	90	
**HEV Ig-M**			
Positive	2	1.8	
Negative	108	98.2	

**Table 2:** Prevalence of anti-HEV IgG and Ig-M antibodies in 90 HIV patients in relation to demographic and clinical data

Characteristics	HEV Ig-G positive N (%)	HEV Ig-G negative N (%)	P-value	HEV Ig-M positive N (%)	HEV Ig-M negative N (%)	P-value
**Gender**						
Male	3(5.9%)	48(94.1%)	0.15	1(2%)	50(98%)	0.71
Female	8(3.6%)	51(86.4%)		1(1.7%)	58(98.3%)	
**Age groups**						
≤ 10	0(0%)	24(100%)	0.01	0(0%)	24(100%)	0.18
20-Nov	7(10.2%)	62(89.8%)		1(1.5%)	68(98.5%)	
>20	4(23.5%)	13(76.5%)		1(5.9%)	16(94.1%)	
**Splenectomy**						
Yes	5(10.2%)	44(89.8%)	0.59	1(2.9%)	48(97.1%)	0.69
No	6(9.8%)	55(90.2%)		1(1.6%)	60(98.4%)	
**ALT (IU/L)**	43.3±32.7	19±19.7	0.001	134±11.3	19.4±16.4	0.001
**AST (IU/L)**	25.7±21.8	18.6±11.5	0.05	69.5±21.9	18.4±10.9	0.001

## 4. Discussion

The transfusion of frequent blood products could be alternative route for cause different disease by infectious agent such as hepatitis C and hepatitis B. Also this condition in these individuals could be leads to physiological problems such as liver injury. Of course this programmed injection in these patients such as hemophilia and thallesemia is critical because the risk of viral hepatitis arising from their needs to blood products. On other hand, there is higher risk of transmission through transfusion for these patients in developing regions than other locations, therefore is very threatening. Based on these possibilities, there are different reports based on parenteral HEV exposure with transmission through transfusion in endemic and nonendemic areas. Therefore the prevalence of HEV antibodies in high risk patients in each region for preventing of high therapy costs is critical.

In our study, the rate of HEV Ig-M and Ig-G antibodies in thallesemia patients were 1.8% and 10%. Previous reports have demonstrated a variety of seroprevalence rates of HEV infection in high risk patients in different regions of Iran (Elizee et al., 2013; Morteza et al., 2009; Taremi, Khoshbaten, Gachkar, Ehsani Ardakani, & Zali, 2005). The seroprevalence of antibody against HEV in thallesemia patients of other parts of world were 0.4%, 2.4%, 10.7% (Al-Fawaz et al., 1996; Hossein Keyvani, n.d.; Psichogiou et al., 1996). One of the studies followed active HEV infection by detection of Ig-M antibody that no individuals was found positive for Ig-M but in our report two of the patients have active HEV infection. In several studies the rate of seroprevalence of Ig-G antibody in other individuals such as blood receipts and donors in different parts of world were performed (Ahmad et al., 2011; Gao et al., 2004; Matsubayashi et al., 2011; Ma et al., 2015). Also the total seroprevalence of IgG antibody in Iranian blood donors in different regions were 4.5% in Tehran 2009 (“Seroprevalence of anti-HEV and HEV RNA among volunteer blood donors and patients with Hepatitis B and C in Iran,”), 5.5% and 8.5% in Jahrom (“Hepatitis E virus and serum level aminotransferases in blood donors,”; “Seroprevalence Of Hepatitis Virus In Blood Donors”), a town of the southern of Iran, 14.3%, urban and rural areas of central province of Iran (Ehteram et al., 2013). Therefore, our data shown that the rate of Ig-M and Ig-G antibodies in Jahrom, southern of Iran, was higher or lower than other parts of Iran and world of course this rate was similar to other reports. There were different approaches in the cases of various seroprevalence in over world. Noting reasons such as demographical and geographical differences in according to academic and developing regions could be varied incidence of HEV infection (Aggarwal, & Naik, 2009). In addition to major transmission route of HEV infection, oral-fecal, this seems to be a probably reason that repeated transfusion effects on getting HEV infection therefore these individuals may be prone to HEV infection. Another possibility is that individuals with anti-HEV Ig-G might have been transferred passively by transfusions and defect expression of acquired immunity. On other hand, the importance of HEV infection in relation to blood multi-transfusion suggesting increased the possibility that the virus could be parenterally exposure or a possibility of combined route of transmission.

Because the HEV infection is hepatotropic, therefore HEV could be the leading cause of acute viral hepatitis (Bhatia, Singhal, Panda, & Acharya, 2008). In our study, liver enzymes levels such as ALT and AST in patients with positive anti-HEV was higher than anti-HEV negative patients. However our and previous studies shown that elevated ALT may be due to sub-clinical HEV infections (Gao, Peng, Zhu, Sun, Zheng, & Zhang, 2004). Therefore in patients with Unexplainable elevated ALT and AST, hepatitis E as an alternative diagnosis is reasonable. In recent study, thalassemia patients may represent the convalescent phase of a previous exposure and potentially able to cause transfusion associated hepatitis E. The presence of patients with double positive antibodies (Ig-M and Ig-G), who ALT levels were higher than HEV single negative subjects indicated that HEV-associated hepatitis due to transfusion transmission may be occur but cannot be deduced from the higher levels of double antibodies and high ALT levels.

In the current study, a significantly trend of seropositivity associated with increasing age between 11-20 years was observed but decreased in over 20 years old. The seroprevalence in other studies among individuals in HEV endemic and no endemic regions was significantly increased with age. They reported that HEV seroprevalence increased with age from 0.9% in children aged 6-9 years to 8.1% in people over 50 years old (Ataei et al., 2009). This condition may be due to an epidemic form of HEV infection which happened in some decades ago, when the sanitary conditions in our country were poor. Generally, potential transfusion of HEV-specific antibodies to the individuals of multiple blood such as thalassemia patients and rising seroprevalence to HEV in over years old and also significantly decreased risk of after-transfusion HEV infection.

In conclusion, this study shows a relatively prevalence of anti-HEV in high risk groups, especially thalassemia patients. Also we thought that an asymptomatic infection of HEV may have lead to the elevated ALT and AST levels in our patients and may have been due to HEV-associated hepatitis. Therefore more studies using polymerase chain reaction techniques are required to confirm whether HEV infection through blood or transfusion is possible. Furthermore, more study are needed to assess HEV screening of blood products to these patients that those have a probably risk of exposure to HEV especially in higher years old.
